# Modeling digital health systems to foster interoperability

**DOI:** 10.3389/fmed.2022.896670

**Published:** 2022-08-19

**Authors:** Frank Oemig, Bernd Blobel

**Affiliations:** ^1^IT-Consultant in Healthcare, Mülheim, Germany; ^2^Medical Faculty, University of Regensburg, Regensburg, Germany; ^3^eHealth Competence Center Bavaria, Deggendorf Institute of Technology, Deggendorf, Germany; ^4^First Medical Faculty, Charles University Prague, Prague, Czechia

**Keywords:** information model, interoperability, data exchange, data set, communication standard

## Abstract

Information systems are a complex thing, and they are mostly not used stand-alone anymore. In that context, many different issues must be considered. It starts with defining the system's purpose, includes the use cases and scenarios in combination with the necessary data ideally separated into distinct domains. Furthermore, it requires the selection of an appropriate set of supporting components/tools and a development environment including some technology to enable continuous integration. And the endeavor does not come to an end with the development of the system itself. To manage those challenges, thinking about design and architectural principles becomes a mandatory element. The situation gets more complicated with growing expectations regarding communication and cooperation between the more and more complex and dynamic ecosystem's actors. The resulting information system has to adhere to different, sometimes contradictory principles and requirements, frequently controlled by different authorities. This paper focuses less on developing information systems in general but concentrates on the aspects that must be considered when multiple requirements from different stakeholders for data exchange and knowledge sharing for advanced interoperability must be met. The latter is commonly underspecified due to missing proper verification of the correct interpretation of data. One intent of the paper is to promote the deployment of information models as a common basis to derive data exchange specifications establishing advanced interoperability. However, it also addresses the necessity to guarantee that the information models and implementable artifacts correctly represent the intended functions and objectives as well as the underlying concepts of the business system in its prevailing context. Therefore, we cannot limit our considerations on the data and information viewpoints.

## Introduction

To enable and facilitate interoperability among applications, many data exchange standards for different purposes such as HL7 version 2.x (1), HL7 Version 3.0 (2), HL7 CDA (2), HL7 FHIR (3), DICOM (4), openEHR/EN13606/archetypes (5, 6), EDIFACT (7), ebXML (8), xDT (9, 10), H.PR.I.M. (11), PN13 (12), NCPDP (13), X12 (14), ClaML (15), CTS2 (16), SNOMED CT (17), etc., have been developed by different Standards Developing Organizations (SDOs). Despite structural and functional differences, they share the same design elements and underlying basic principles such as specific topologies, the Open Systems Interconnection model (17), etc., which will be following explained in more details (18). Nevertheless, most of those standards lack the same foundational basis – a data model resp. an information model.

A data model is sometimes mixed up with data structures or database models, although it organizes elements and defines how they relate to each other. In most cases, they should refer to real-world entities even though the interpretation of that is left to the reader. According to Lee, an information model is “a representation of concepts, relationships, constraints, rules, and operations to specify data semantics for a chosen domain of discourse” (19). Therefore, an information model needs a sophisticated formal representation. This paper especially focuses on the formalization of a model to enforce a correct interpretation of requirements controlling the system's development and implementation. Thereby, it follows the good modeling best practices described, e.g., by Langhorst et al. (20), especially considering the characteristics and increasing constraints of representation styles and languages throughout the development process, that way guiding justification and transformation of the different models. In other words, the paper adds theoretical and general linguistics as well as systems theory to the traditional way of discussing development processes.

Information Communication Technology (ICT) builds the foundation for data exchange. This paper focuses on the ICT perspective of interoperability in transformed health ecosystems represented according to the ISO 10746 Open Distributing Processing Reference Model (RM-ODP) (18) and its extension according to ISO 23903 Interoperability and Integration Reference Architecture (15). After roughly introducing and exemplifying the ICT-specific viewpoints (VPs) Enterprise VP, Information VP, Computational VP, Engineering VP and Technology VP, it especially elaborates on commonalities, differences and their relationship as a solid foundation. Furthermore, it focuses on the data sharing interoperability paradigm with its logical information models and associated implementable technology specifications. A second aspect of this paper is to demonstrate and explain the foundational equivalence of different standards, that way demonstrating the importance of information models as a common reference to achieve interoperability.

## Viewpoints

Information Systems have to follow a well-defined life cycle, including design, development, implementation, test, and in some cases certification. According to the reference model for open distributed processing (RM-ODP) (18), these process steps can be aligned with different viewpoints which are roughly introduced as follows.

### The enterprise view on digital health

The Enterprise View takes care of the business processes in a specific environment. It describes the IT-specific use cases and workflows that should be managed within – or across – organizations with a focus on purpose, scope and policies of the system. Commonly, transaction and interaction models explain how different (abstract) actors are working together and interacting to achieve a common goal. They are represented in common notation forms such as Business Process Markup Language (BPML, BPML+) and Business Process Model Notation (BPMN) (21).

### The information view on digital health

The Information View defines the informational components of the system and their relationships in form of (domain) information models, expressed in class diagrams. It focuses on the semantics of information and information processing performed. The data/information is coupled with the processes described in the previous VP.

These viewpoints together are going to exemplify interaction and information models. The Unified Modeling Language (UML) (22) is one possible, but the most commonly applied formalism that can be used to express those diagrams.

### The computational view on digital health

The Computational View enables the distribution of the system by decomposing it into objects according to structural and functional requirements, including necessary interfaces. This view concentrates on the computation to be performed.

### The engineering view on digital health

The Engineering View takes care of the design of the information system under consideration by defining and interrelating implementable artifacts.

### The technology view on digital health

Finally, the Technology View focuses on the specific technologies like IDEs, programming languages, libraries, etc., chosen to implement, run and maintain the system.

### The business view on digital health

When designing digital health systems, we must always have in mind that the requirements for the solution as well as the relevant concepts have to be defined by experts from the domains involved in the business system and the business process use cases. The concepts must be formally represented using those domains' ontologies. Therefore, the viewpoints of ISO 10746 must be extended by another, ICT-independent view – the Business View - as defined in ISO 23903 (see Chapter 9.2), which thereafter has to be transformed into the aforementioned views. The Business View guides all the other view (23). The Business View guides all the other views.

## FHIR as a new foundational standard

One of the standards mentioned in the introduction is extraordinary and therefore worth a more thorough investigation during the course of this paper: the Fast Healthcare Interoperability Resources (FHIR) specifications (3). FHIR is the newest product family of HL7 International and currently transitioning from phase 1 (technology trigger) to phase 2 (peak of inflated expectations) according to Gartner's hype cycle. Furthermore, FHIR realizes a boot-strapping process, therefore allowing a self-definition using its own representation form. Finally, it is the foundation for a set of other standards and developments like CDS Hooks, “Smart on FHIR,” etc.

**Figure 1:**
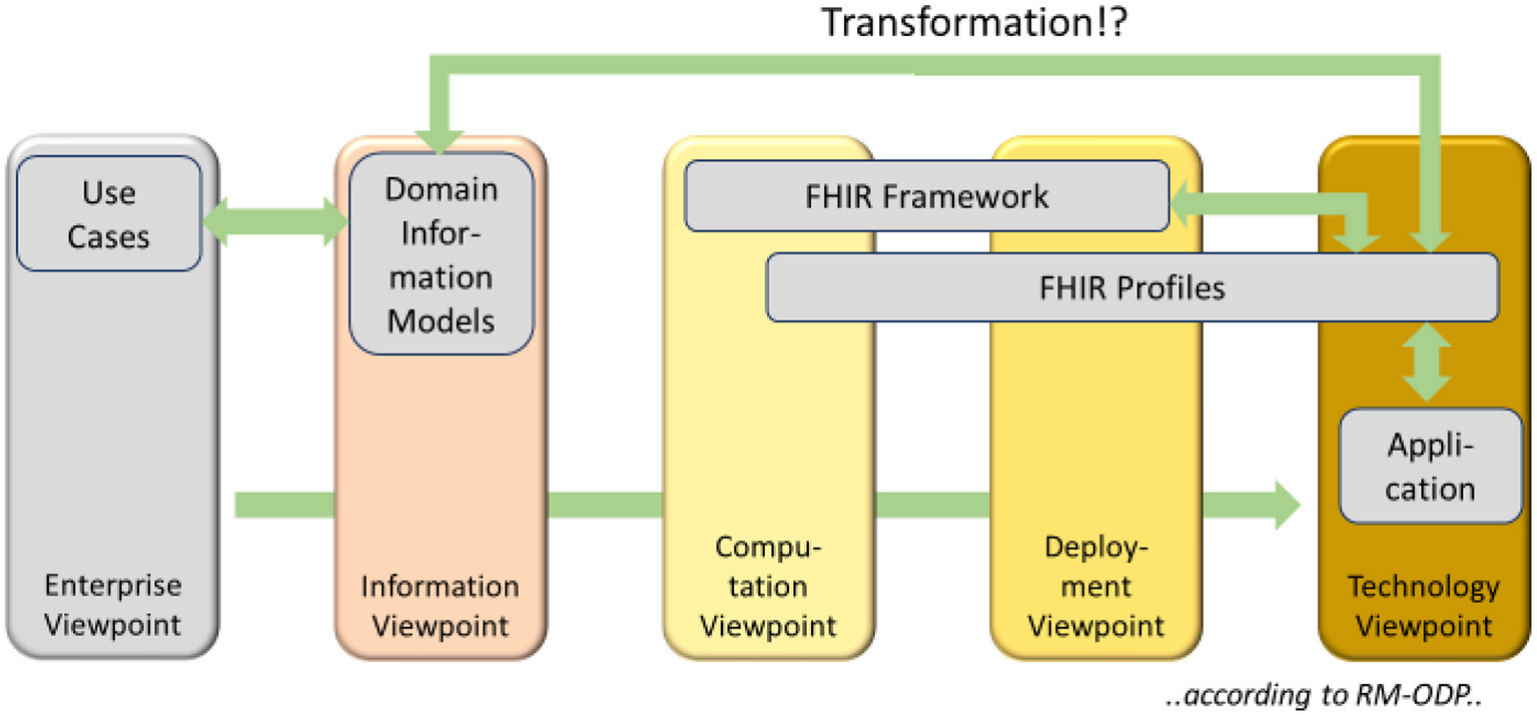
FHIR Framework (3) according to RM-ODP (18).

FHIR as a foundational framework and implementable artifacts must be placed across the computational, engineering, and/or technology viewpoints, depending on what is judged (Figure 1). The easiest part is the technology, because FHIR is based on XML and/or JSON. Therefore, information systems must create FHIR instances in one of those representation forms.

The resources as defined by the FHIR framework belong to the computational and the engineering viewpoint, respectively, as they specify how the information must be separated and spread into certain components (engineering), and what kind of functionality they support (computational). FHIR profiles establish the semantic bridge to the information models because they define the semantics that is represented by specific FHIR resources in their use-case specific deployment. Therefore, FHIR profiles mostly belong to the technology viewpoint because technical aspects are described and specified in detail, but profiles also span over to the computation and engineering viewpoint due to their foundation.

### Communicating data

**Figure 2:**
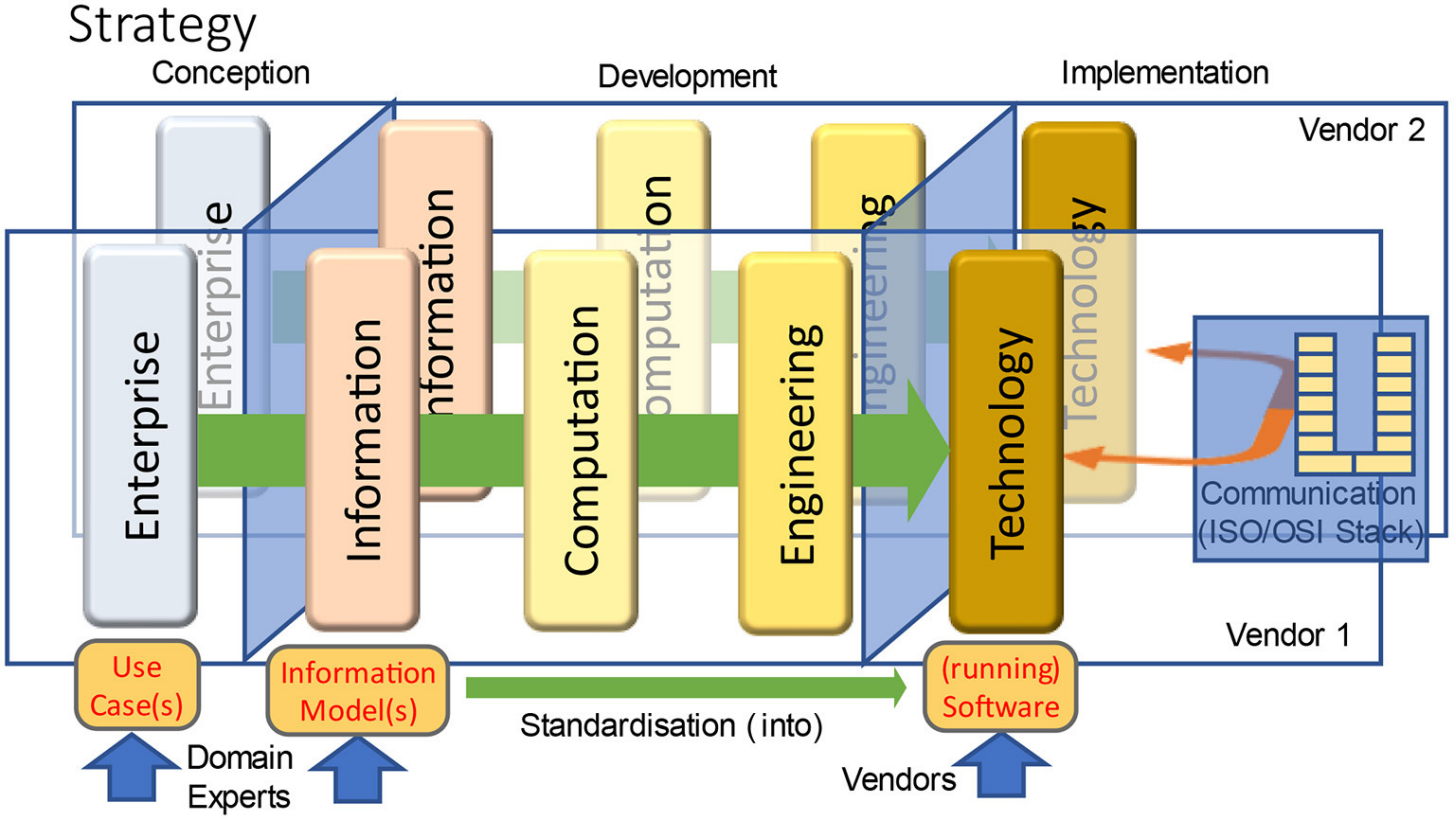
RM-ODP (18) and ISO/OSI-Stack (24, 25).

Although RM-ODP is designed to support the development of information systems, it does not contain an explicit viewpoint in Figure 1 for expressing communicating systems even if each system has been designed according to the principles listed before. According to Figure 2, the aspect of two or more information systems sharing their data can be best represented and expressed in the technology view. They have to communicate according to the ISO/OSI-stack (26). In Chapter 8 (**Figure 15**), it is explained in which way this aspect is influencing interoperability and therefore reusability of data in form of information. The way the different viewpoints interact can be best seen in (27).

## Designing and managing information models for interoperability

**Figure 3:**
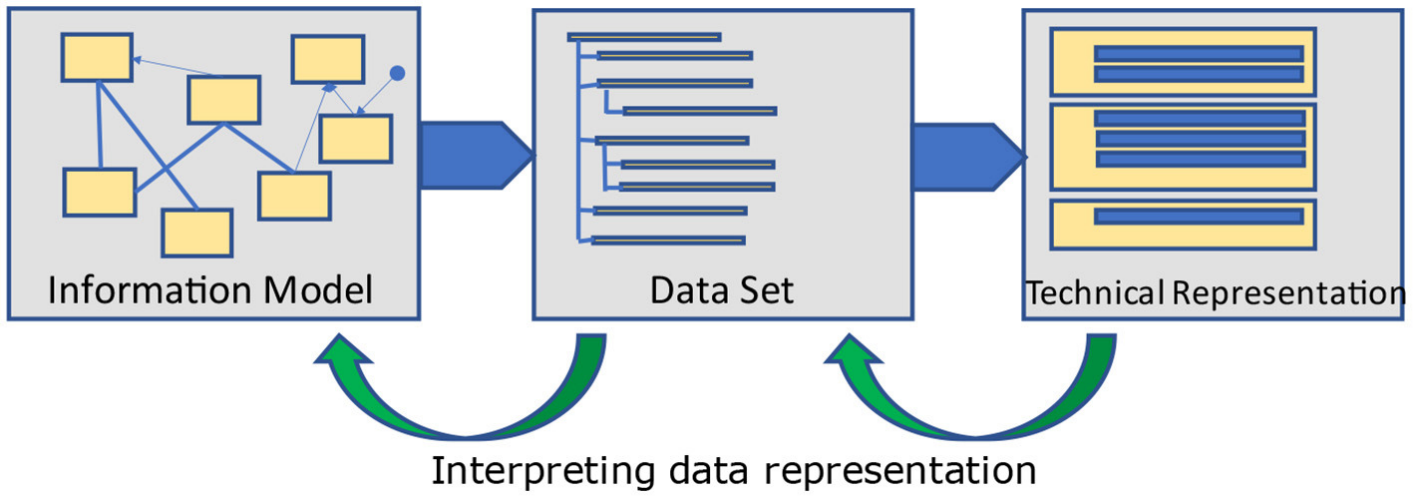
Derivation process.

Information models are built as entities with relationships among them. These relationships lead to closed loops - cyclic graphs. Frequently, the opinion dominates that information models as cyclic logical models are too complicated and therefore unnecessary for data exchange, and requirements of healthcare providers are adequately covered by data sets as a hierarchic definition of data elements alone. Therefore, most implementation guides for data exchange only provide hierarchic data element lists – named data sets (Figure 3) and do not introduce references and crosslinks to other information elements which are necessary in complex information systems. However, as long as only representations of forms/questionnaires are requested, the difference between data sets and data models will not show up and is ignored thereof – or at least not seen/realized.

**Figure 4:**
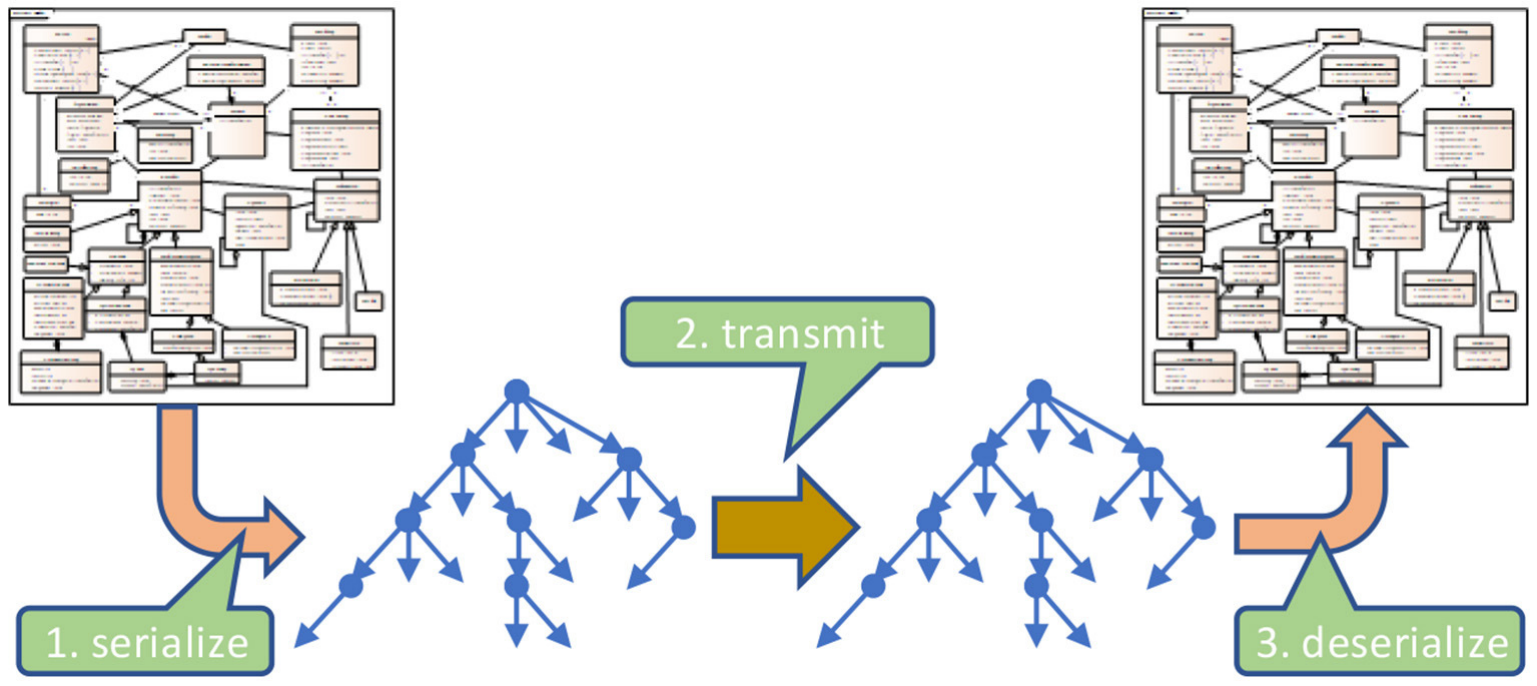
Communication.

For communicating data among different applications, the data set is taken and transferred into a technical, hierarchically equivalent representation that can be implemented – as sender and recipient – with the same or different expectations. Consequently, a sender is taking the data from its storage and converts it into that technical representation, whereas the recipient is implementing the opposite, namely extracting the data from that representation and converting it into its own data storage model (Figure 4). Only a few standards exist, that allow for direct storage using that exchange format, so that this transformation is obsolete. To answer the interoperability question, it must be verified that the underlying models from both the sender and the recipient perspectives adhere to the same information model. If this is not the case, some information details cannot be provided or stored. The loss of information is the consequence. The driving force for data exchange specifications is the information model components and relations implicitly contained within the specification.

**Figure 5:**
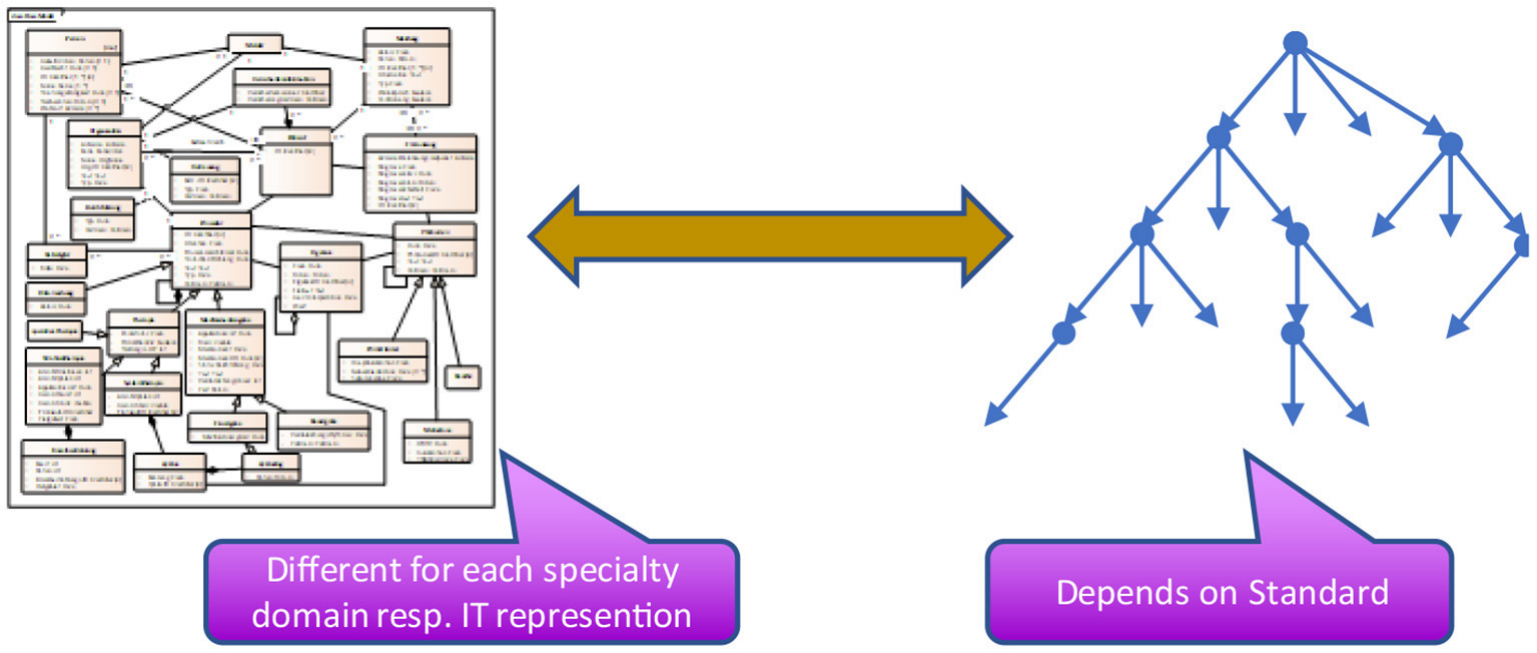
Projection to standards.

It should be clear, that the hierarchic structure of the information objects and the corresponding technical representation depends on both – the underlying information model and the standard that should be used for data exchange (Figure 5). It is necessary to find a bijective transformation from the hierarchical representation into the standard and back again without losing information.

If the standard being used allows for arbitrary variations of the structure, as it is the case, e.g., with XML at the highest level, this bijective transformation is easy because one is free in specifying the entities and attributes according to the direct needs. On the opposite, such a structure is individually created and therefore specific to the needs, so that no reasonable reuse is possible.

If the standard provides a set of dedicated structures, e.g., HL7 V3 or FHIR, the transformation is more complicated, because an alignment or mapping is needed. When the number of models resp. hierarchic data sets is greater than the number of available structures, certain marks must be established that can be used to identify what data set is represented. In high level data exchange standards like HL7 v2, V3, CDA or FHIR, these available structures are called profiles or templates.

### Details

**Figure 6:**
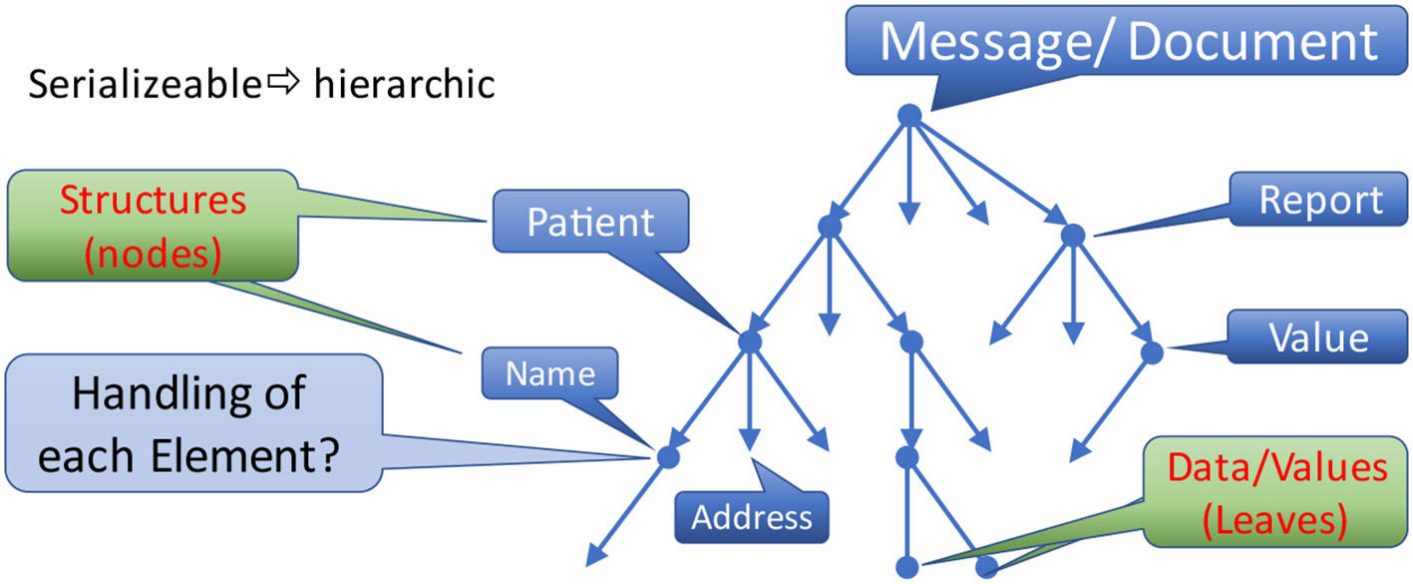
Hierarchic data structure.

Communication scenarios are using one of two possible exchange paradigms: sending messages or documents. The first conveys information triggered by events for data processing and storing, discarding the original message afterwards. The second transmits a complete set of information elements for storage as a whole accompanied by metadata specifying the context. Independent of the paradigm, both facilitates a hierarchic tree-like structure, beginning with the message context or the root element of the document (Figure 6). All branches of the tree represent appropriate parts.

What attributes do the nodes with substructures or leaves provide?

The nodes of this tree enforce the overarching structure. For example, a message/document contains information about a patient. The patient data consists of his name and address. The name is comprised of first and given name as well as other details. In the end, real data is only provided with the leaves of this hierarchic structural representation, as non-terminal elements are simply introduced to group them for control purposes.

**Figure 7:**
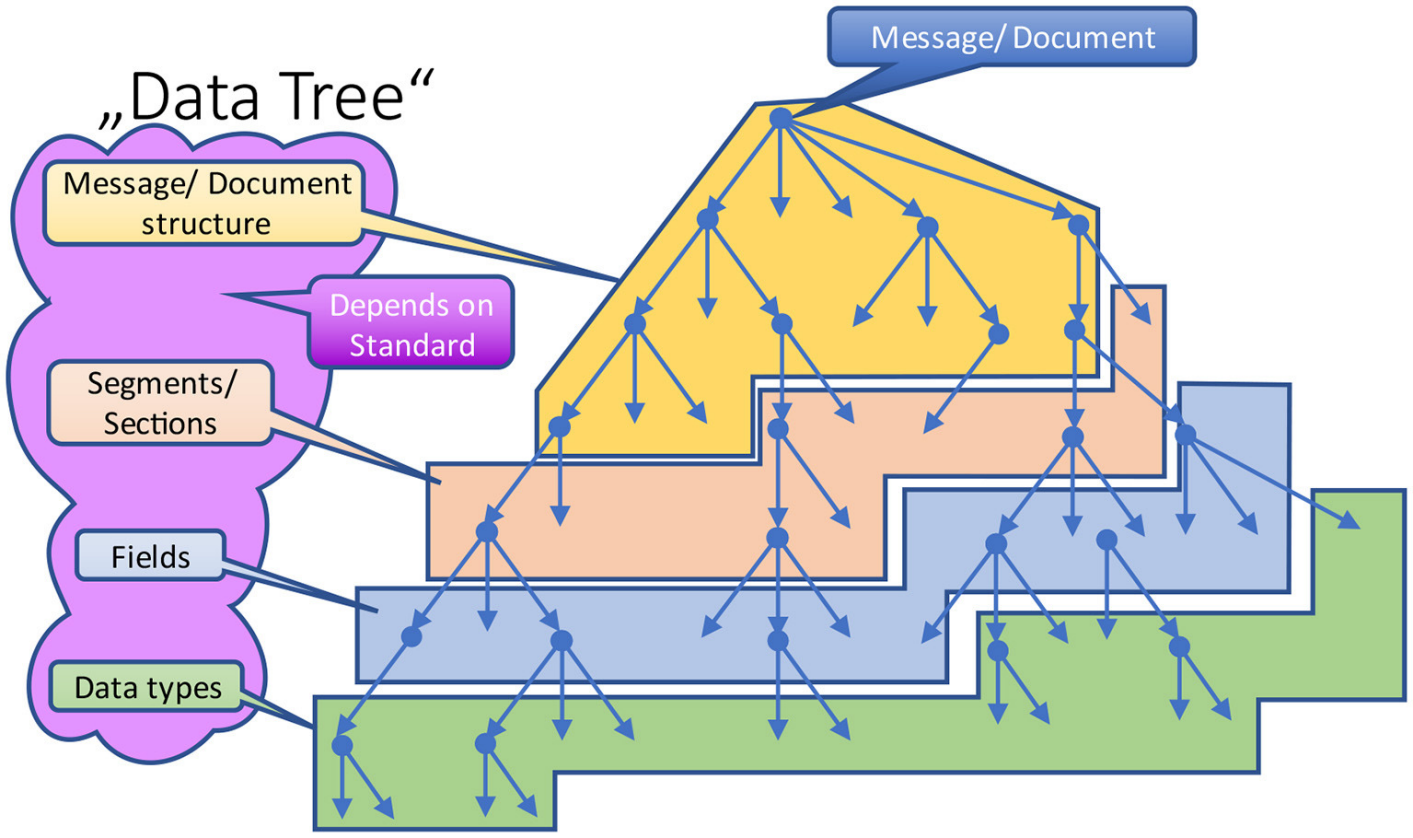
Abstract data tree.

Depending on the paradigm and the underlying communication standard, the naming of the different branches (levels) varies (in Figure 7 marked by different colors). For example, a message is comprised of segments and fields, whereas a document contains sections and subsections. Some standards distinguish between (logical) data structures and (technical) data types. In the end, they all contribute to this data tree in form of different levels (Figure 7), and it does not matter how many individual levels are defined and what their technical purpose is. From their attribution, they obey the same rules.

**Figure 8:**
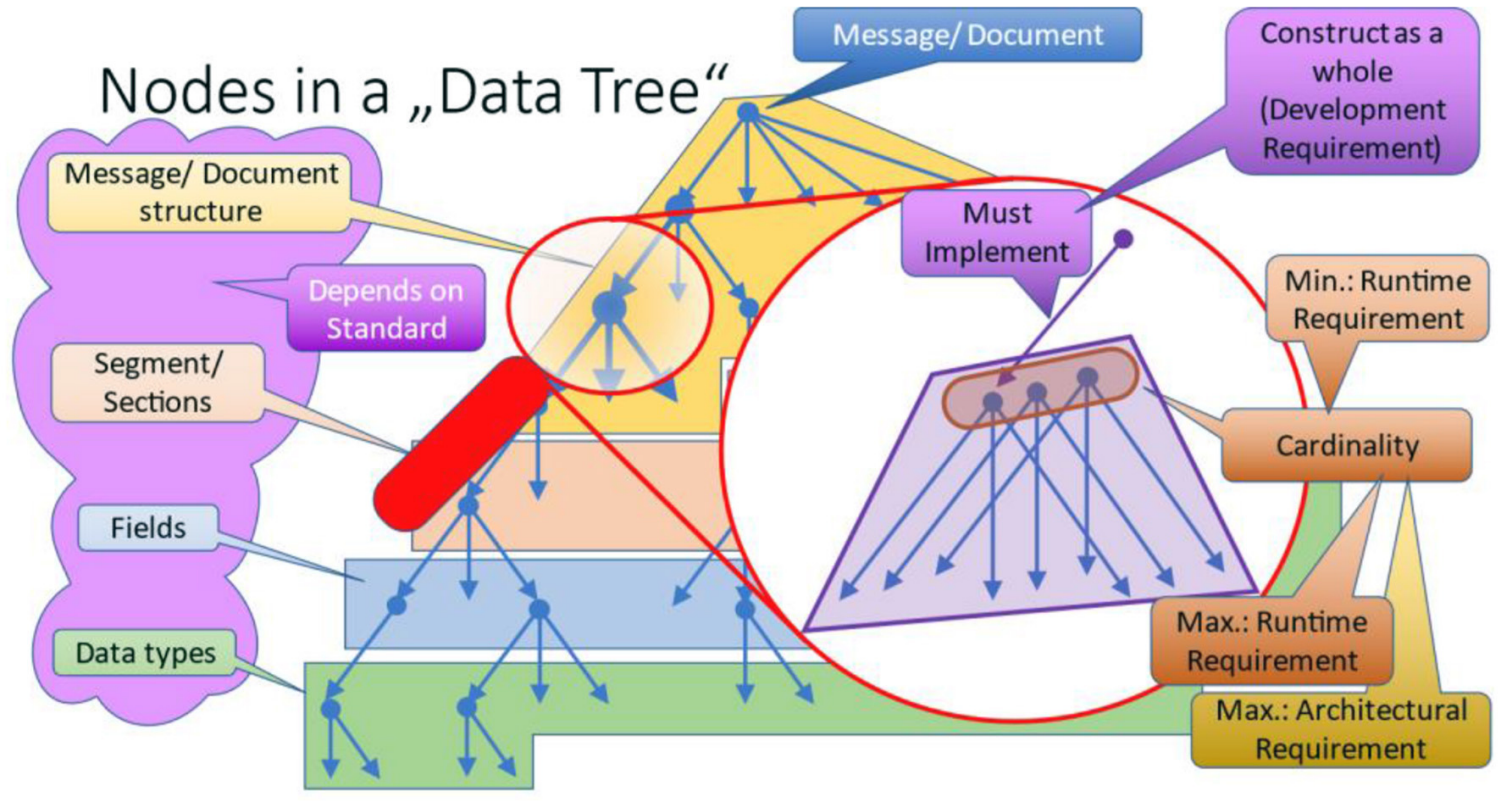
Nodes in a data tree.

The interesting part are the non-terminal nodes within that hierarchic structure. In Figure 8, one node is enlarged to explain the internals.

**Figure 9:**
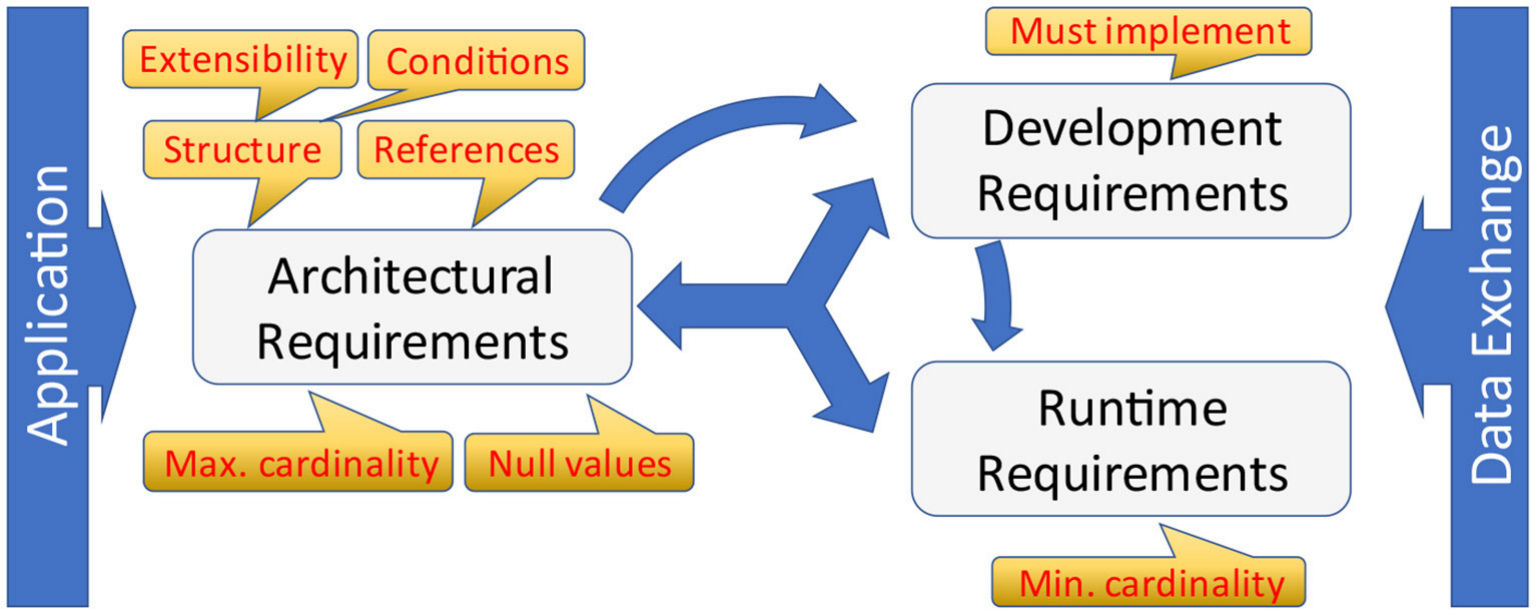
Architectural, development and runtime requirements.

The nodes conceal architectural, development and runtime requirements (Figure 9). Unfortunately, data exchange specifications only express development and runtime requirements explicitly so that those can be considered during consecutive specification processes. The names of those constructs vary and may occur in different pre-coordinated types, e.g. “optionality,” “must implement,” “must support,” “required,” “mandatory,” “repetitions,” “cardinality,” and some more.

Architectural requirements have an impact on structure and general capabilities of an application itself. The most obvious aspects are the structure as introduced in form of logical groups repeating those structural elements, and links to other sub-structures. The latter introduces cyclic graphs within the underlying information models. Extensions and null-values are two further architectural aspects that are often not interpreted as such. Both impact the design of applications, because the capability to store additional, most probably unexpected information or information about the absence of data is a central challenge for the basic design of an application, esp. for data storage. In this context, two standards must be mentioned:

a) HL7 Version 3/CDA (2) is the only standard that has a built-in capability for null-values for all data elements and attributes. Due to the underlying architectural framework, extensions are not directly allowed, or only within implementation guides facilitating a different namespace.
b) HL7 FHIR (3) allows for extensions at every part of a data instance. That opens the door for all kind of variations including different ways of conveying null-values (reasons for missing/absent data). Developers are challenged to consider these architectural requirements within their information systems.

Another interesting aspect is conditions (often also called predicates) that describe inter-dependencies among different nodes. As supporting a node is an architectural requirement, evaluating and supporting conditions is such a requirement as well.

**Figure 10:**
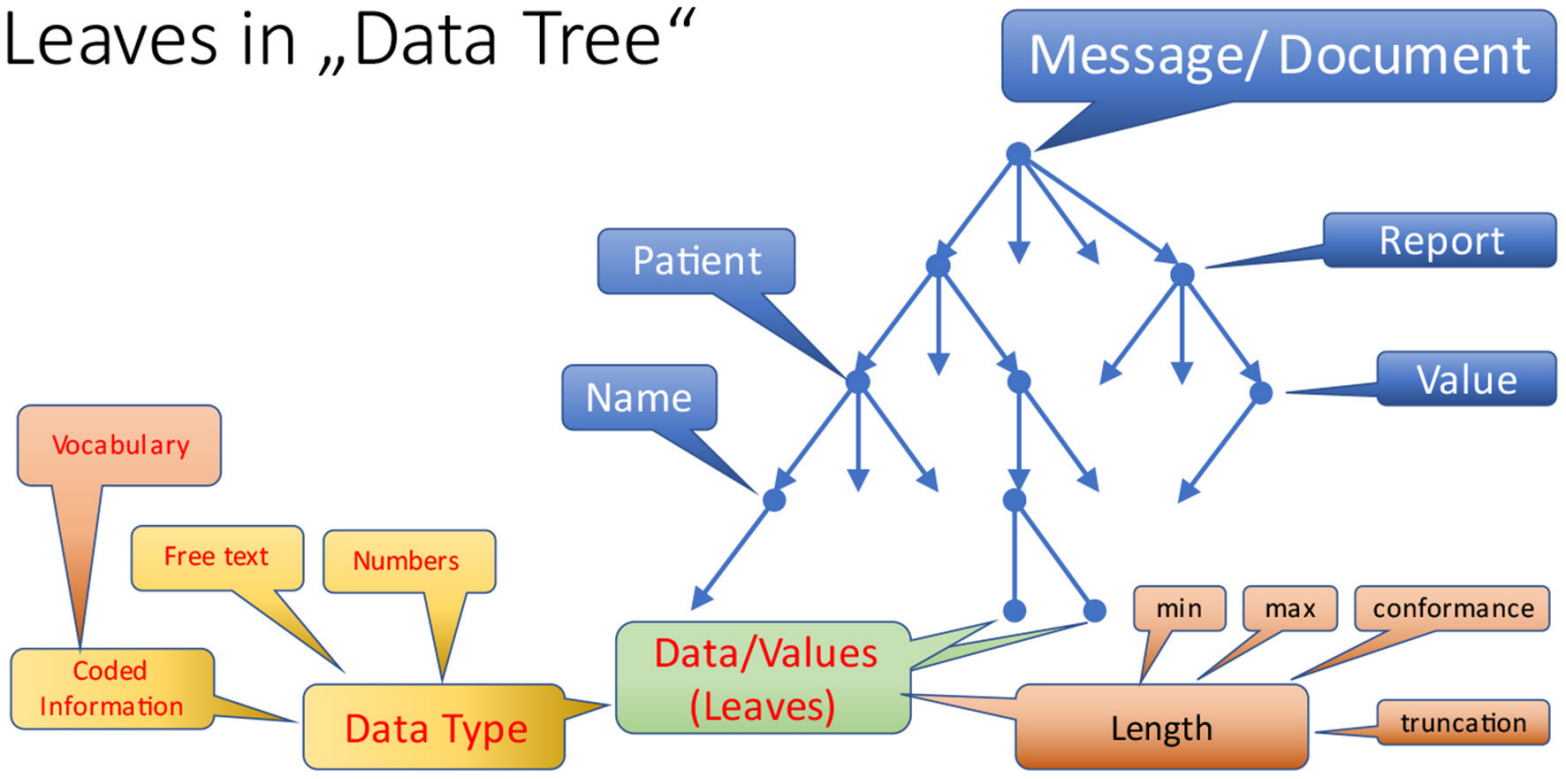
Leaves in a data tree.

In contrast to the nodes, leaves add even more requirements, because they are responsible for managing and maintaining particular values for data elements (Figure 10). So, different data types primarily handle textual and numeric information as well as coded information. The latter must be bound to appropriate vocabulary which is a dedicated and separate topic.

An important aspect for maintaining data items is the length of the value, although most interoperability specifications do not care about this anymore. Minimum and maximum length only make sense for rare use cases, esp. when the length is a real (physical) restriction, and the content of the use case is clearly defined. To overcome this problem, some standards have introduced a conformance length that informs about a reasonable value for a minimum length. As such, it can be treated as an advice for developers. The absence of length details is quite frequently accompanied by truncating the information during storage, because the target field in a database is too short, and most interfaces do not handle it adequately. In order to eliminate this problem, some standards have introduced a truncation flag, that indicates, in which way truncation is allowed or not. For example, truncating the house number in a street line information is certainly not nice, but cutting off relevant information in coded or textual information may cause severe misinterpretations leading to harm a patient.

### Information model representation

**Figure 11:**
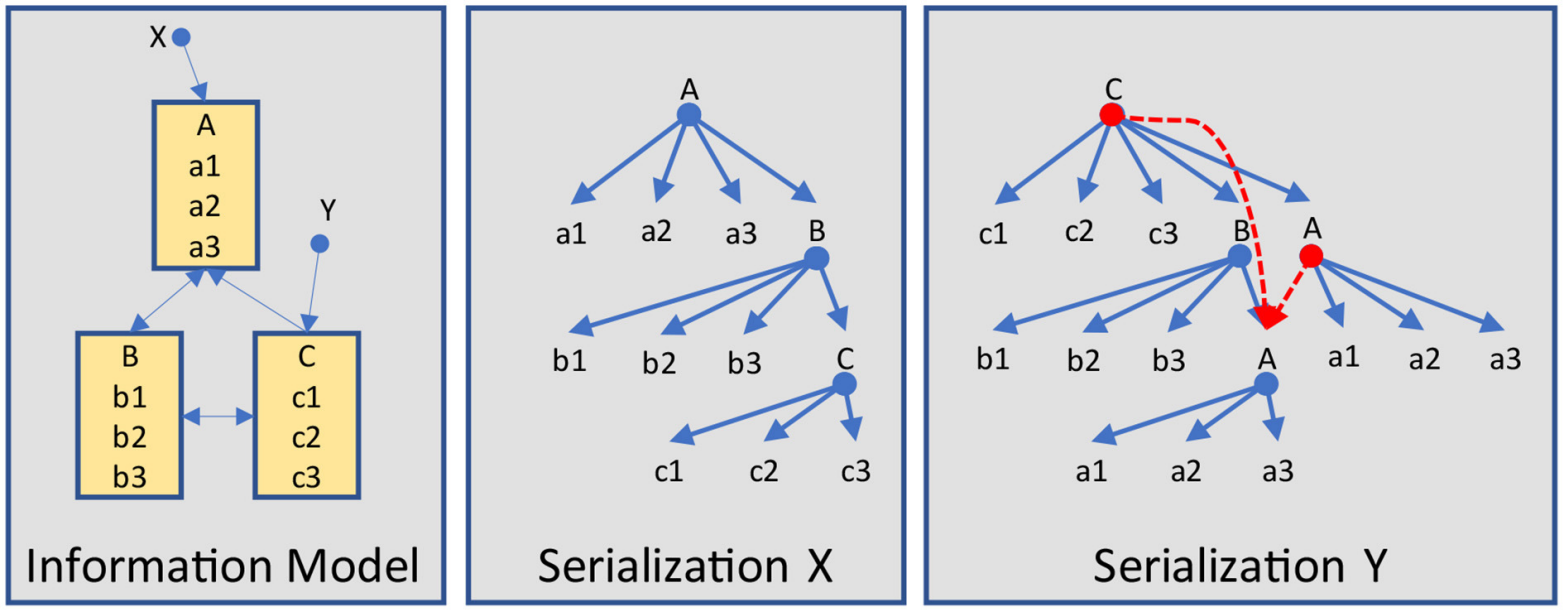
Serialization examples.

As introduced previously, the structure resp. the hierarchy of data elements highly depends on the underlying information model (example in Figure 11), even if none is explicitly defined. As standards developers have such a model in mind when defining certain structures, this problem is not worth a discussion. For example, the family and given name parts are obviously associated with the name and not with an address. And a patient is going to have a name – or several names in the course of time – depending on the necessary details. Consequently, such simple aggregations do not cause major or longer lasting discussions. The same applies to arbitrary structures that are used with forms, which are mainly driven by human readability, so that circular definitions do not occur.

Therefore, information models come into play when different aspects for reporting must be considered, that are taken from different parts of this model and reference other parts as is shown in Figure 11. Other good examples are taken from order entry workflows in combination with reports. In essence, the receiving application must “reconstruct” this model from the data it has received. As explained above, if the structure is not compatible, information loss is the consequence. Hence, explicitly providing the underlying model is the preferred solution against best guesses and implicit assumptions of developers.

Another problem is the handling of references to the same information item, as is demonstrated in Figure 11. Whether the information itself is included in the data, repeatedly represented, or simply referenced, offers different options and requires an explicit definition of how it should be handled.

## Information model handling

**Figure 12:**
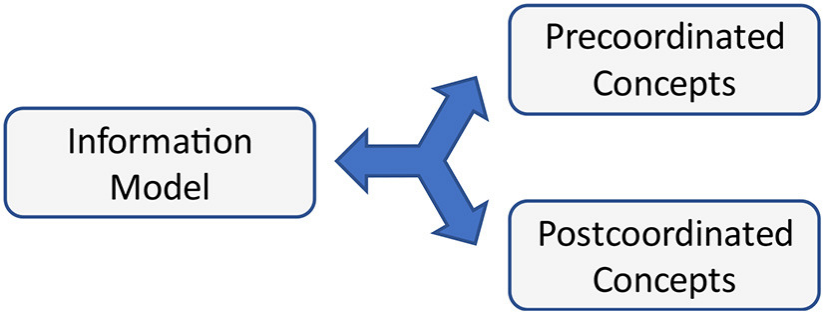
Equivalence of different modeling approaches.

The authors observe strong discussions about using a set of dedicated concept codes instead of defining appropriate information models (Figure 12). Of course, certain details of information models can be pre-coordinated into concept codes. This requires of course that both sides refer to the same coding scheme. Furthermore, codes hide specific contexts, objectives, and perspectives explicitly mentioned in the model and so guiding the interpretation. The deployment of codes only considers and covers the details that can be captured as different axes from a central concept. As an example, the different blood pressure measures allow for aggregating the cuff type, position, load, interpretation, and other details, as they are not exclusive and not repeating. Body weight does not allow for such an approach, because the different forms of amputations would result in a combinatorial explosion of possibilities if expressed explicitly.

Using pre- or post-coordinated concepts is of minor importance because of lossless conversions between them.

The size and assignment of models to a specific domain is of major importance. From a good modeling perspective, the individual information models should be kept small in order to simplify maintenance and promote consistency. Furthermore, they should not bridge different domains, as they might use different information objects for representing the same concept and vice versa. Especially the latter can be seen in many information systems, when clinical, medical, administrative and financial information is mixed, and therefore it is unclear what is exactly represented, and for what purpose it can be used.

## Information models vs. ontological definitions

For correctly and consistently designing and interrelating information models, we have first to understand the concepts and relationships of the business system and its components, representing them using domain ontologies. Thereafter, we must model the business system from its ICT solution perspective at the enterprise level. For that purpose, we must use appropriate techniques and languages like BPMN. The formal representation of concept models using ontologies allows for describing the details in a computable form. Understandable models are an important step forward, as such models are frequently not provided, as mentioned above already. The more one think about contents, the less the semantic details are clear to the reader. In combination with (new) intelligent, knowledge-based techniques like Artificial Intelligence (AI), Machine Learning (ML) or big data, an ontological description becomes necessary. Snomed CT ontology (17) is a good example that demonstrates the possibilities when using the definitions for computation. The authors want to underline and motivate for concentrating on ontological definitions of information models using a computable form. The outcome helps with technical representations for storage and transmission as introduced above to enable advanced interoperability.

## Information models' representation form

**Figure 13:**
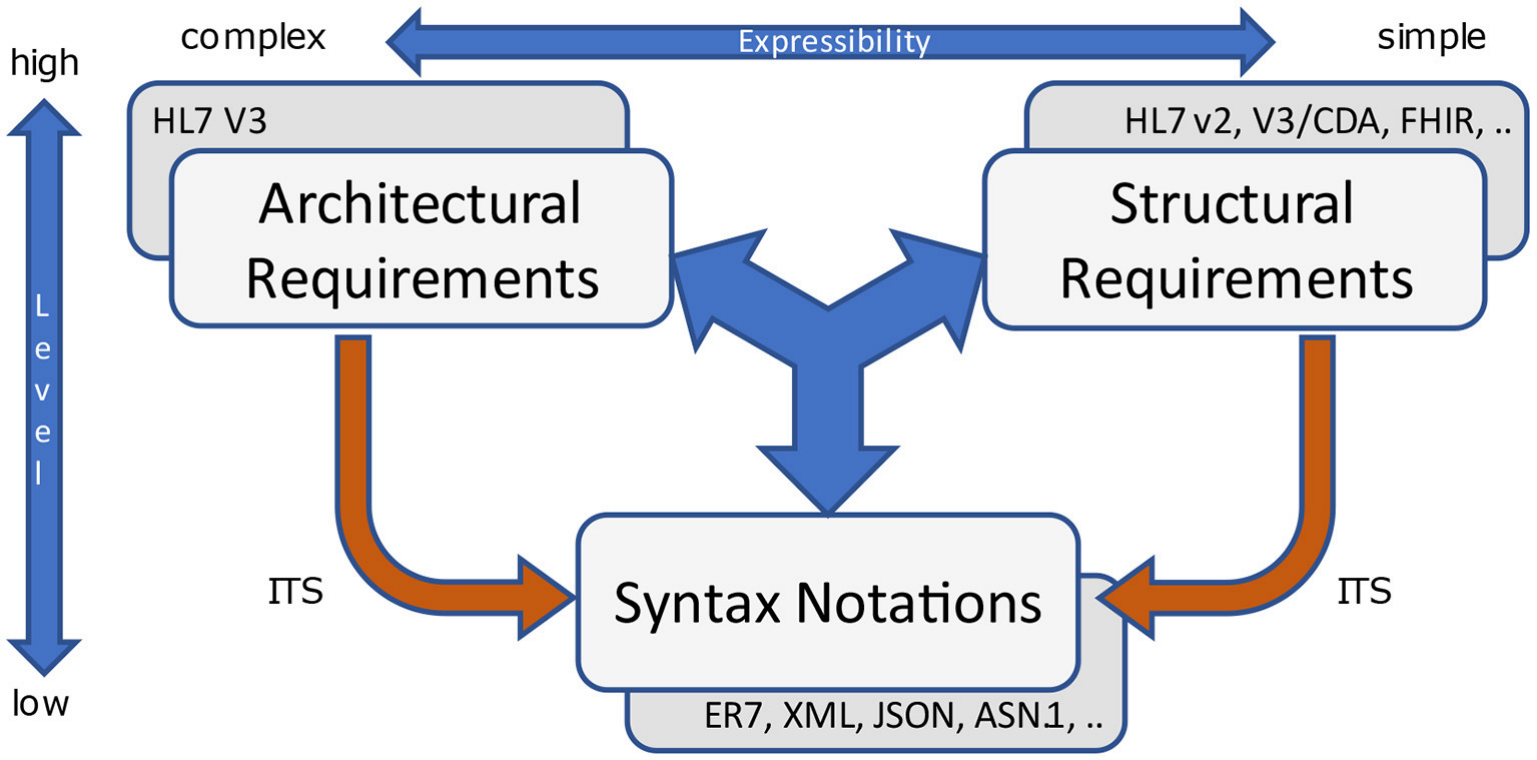
Representation forms.

Another topic worth mentioning is the option for different representation forms. The authors remember a question from the nineties about “what is better, HL7 (v2.x) or XML?”. This question is of course a rhetoric one, and abstracts from the levels that are used for representing data. Furthermore, it hides the disability to distinguish between those levels. Nowadays, modern representation forms are on a higher (ISO/OSI) level and facilitate XML, JSON, ASN.1 and others on the lower level as their implementable technology specification (ITS). Some also allow for bijective transformation between different syntactic representations (Figure 13).

Nevertheless, these aspects do not favor architectural and structural requirements against each other. Both are necessary to develop and implement information systems.

## Information models, standards and applications

**Figure 14:**
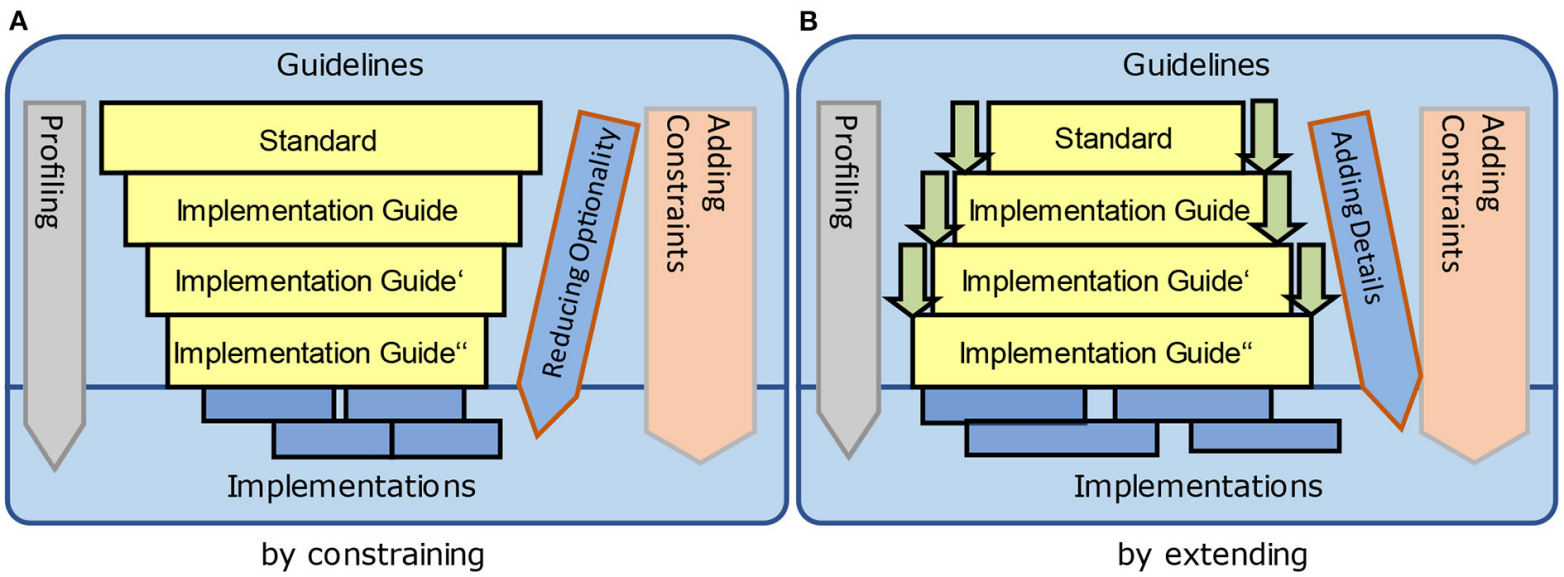
Creating specifications by adding constraints. **(A)** By constraining and **(B)** By extending.

The relationship of information models to applications and data exchange standards has been explained before. Standards that are handled by adding constraints (Figure 14A) impose a greater adherence to the standards from the beginning, whereas adding constraints by extensions (Figure 14B) are easier to manage and to define so that the acceptance is higher. However, the latter does not exclude and avoid additional and new architectural requirements that are not foreseen by the base standard.

**Figure 15:**
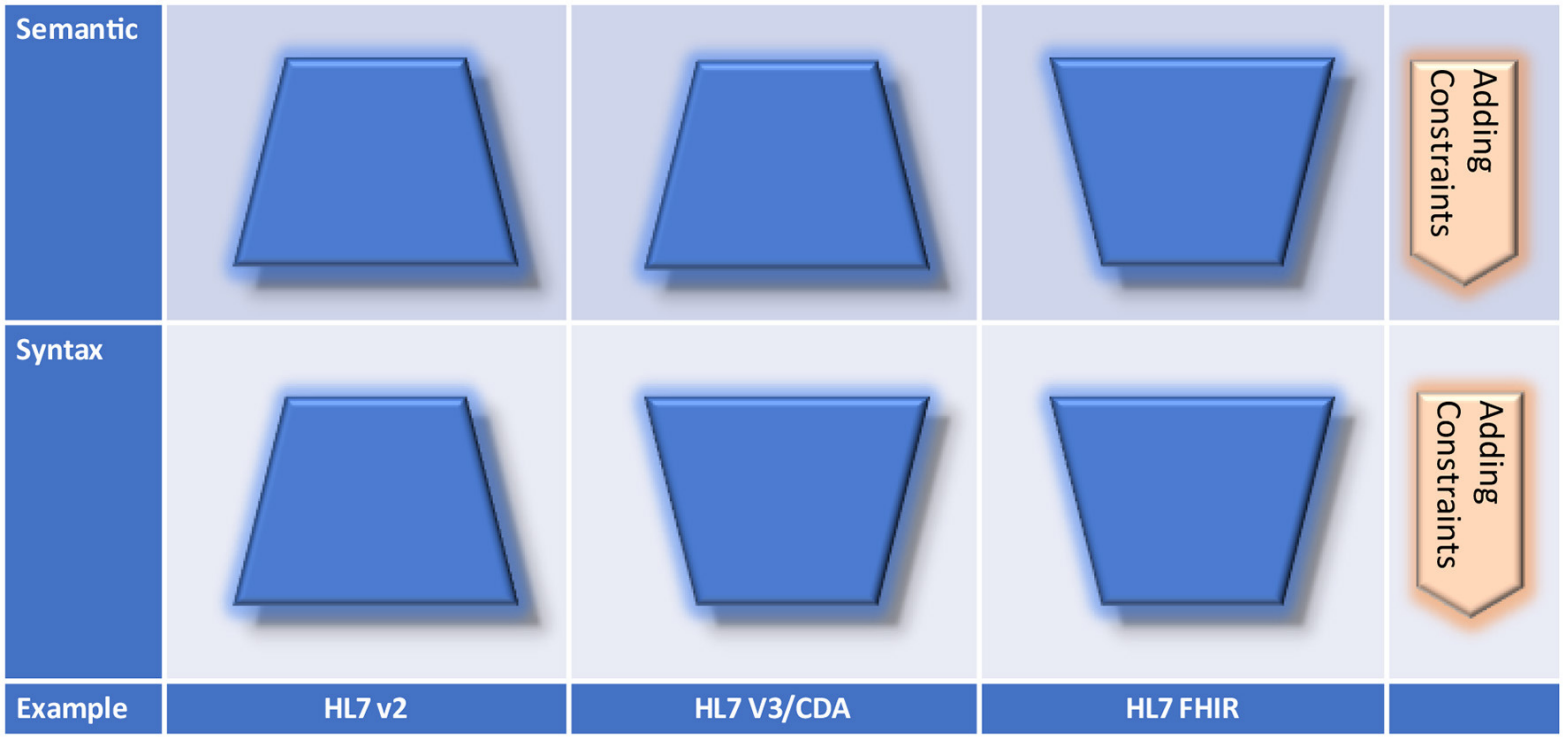
Creating specifications by standards.

The principles for creating specifications according to Figure 14 are realized by specific standards in different practical ways (Figure 15), because the syntactic and semantic perspective must be analyzed separately. HL7 v2.x allows for extending the encoding syntax to add new contents by user-defined segments that must be considered during the parsing process. In HL7 Version 3/CDA, new content can be added by constraining the XML representation. The attribute/value-pair approach for extensions within FHIR is in principle a specific constraint for both – the syntax is closed although the semantics is open.

**Figure 16:**
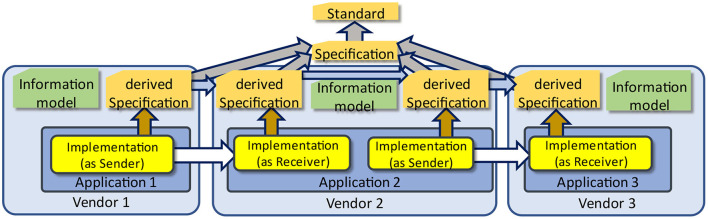
Information models within applications.

Another strong relationship is the use of internal information models as an architectural foundation to the application itself. The structure of the database for storing the information introduces such an information model, although it is not always made explicit. Consequently, a vendor is facing the challenge to convert from its internal structure the external one as defined by interoperability specifications. This challenge becomes even more complex, when a vendor must import data from different other applications, and export it to others as well (Figure 16). As previously explained, if the underlying structures differ and multiple im- and exports according to different data exchange standards are preformed, the probability to lose information is high or at least increasing.

In Figure 16, a communication scenario is presented, where all applications have to adhere to the same base specification. The interoperability challenge for a chain of communicating applications, thereby not losing or misinterpreting information, is even more difficult if the different base specifications and/or base standards have to be used, which facilitate different underlying information models.

### (Improved) Definition of interoperability

Figure 16 also triggers to reconsider the well-known definition of interoperability. IEEE Standard Computer Glossaries (1990) defines “interoperability” as

… “the ability of two or more systems or components to exchange information and to use the information that has been exchanged.”

**Figure 17:**
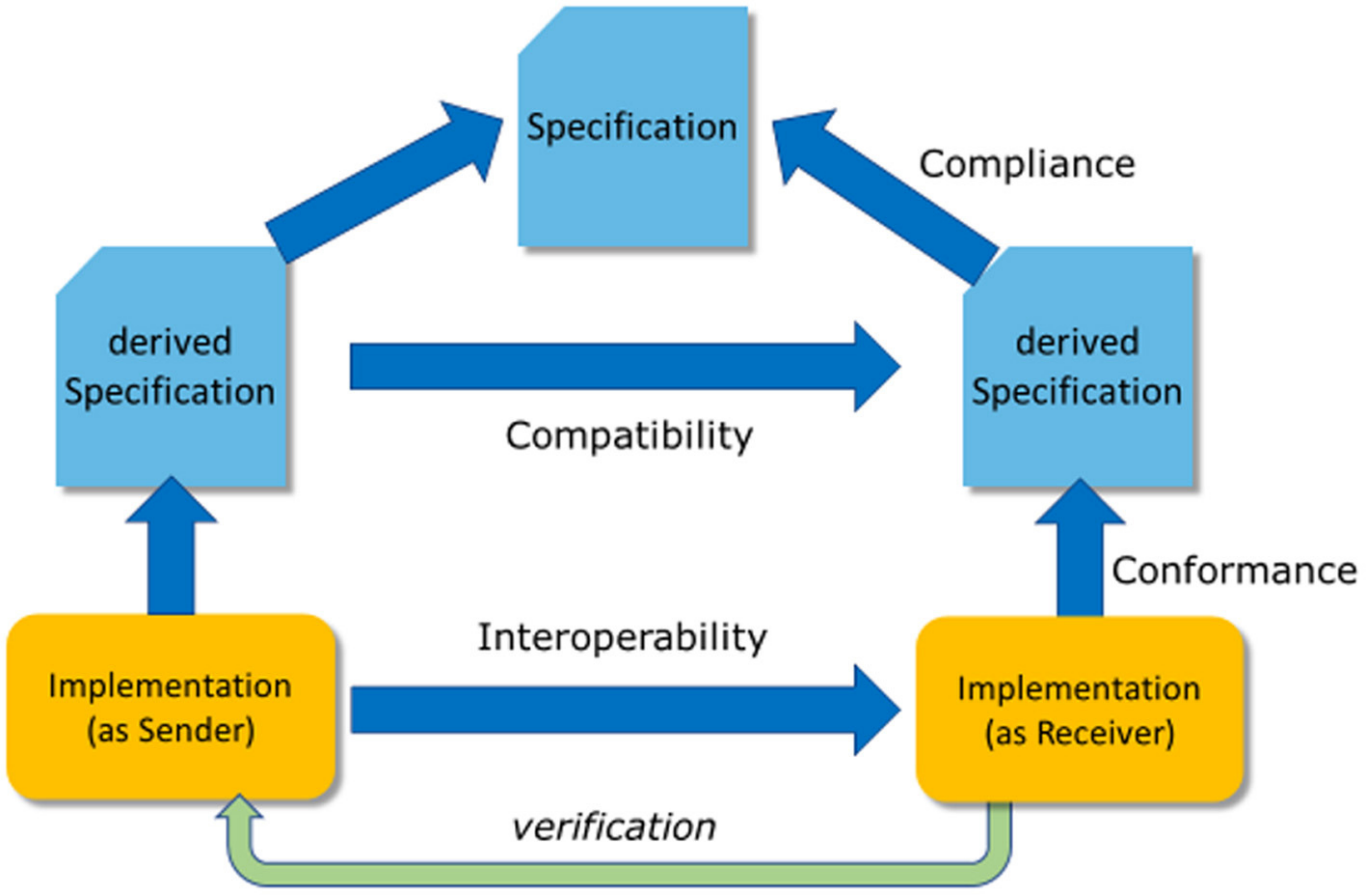
Enhanced definition of interoperability.

Merriam Webster's Dictionary defines interoperability in a military context that associates the concept with using weapons. The latter reveals a hidden condition: This definition lacks an implicit verification that the data has been understood according to the sender's intention, which is symbolized by the green arrow in Figure 17. In other words, there must be some kind of feedback loop to verify the recipient's interpretation against the sender's understanding.

**Figure 18:**
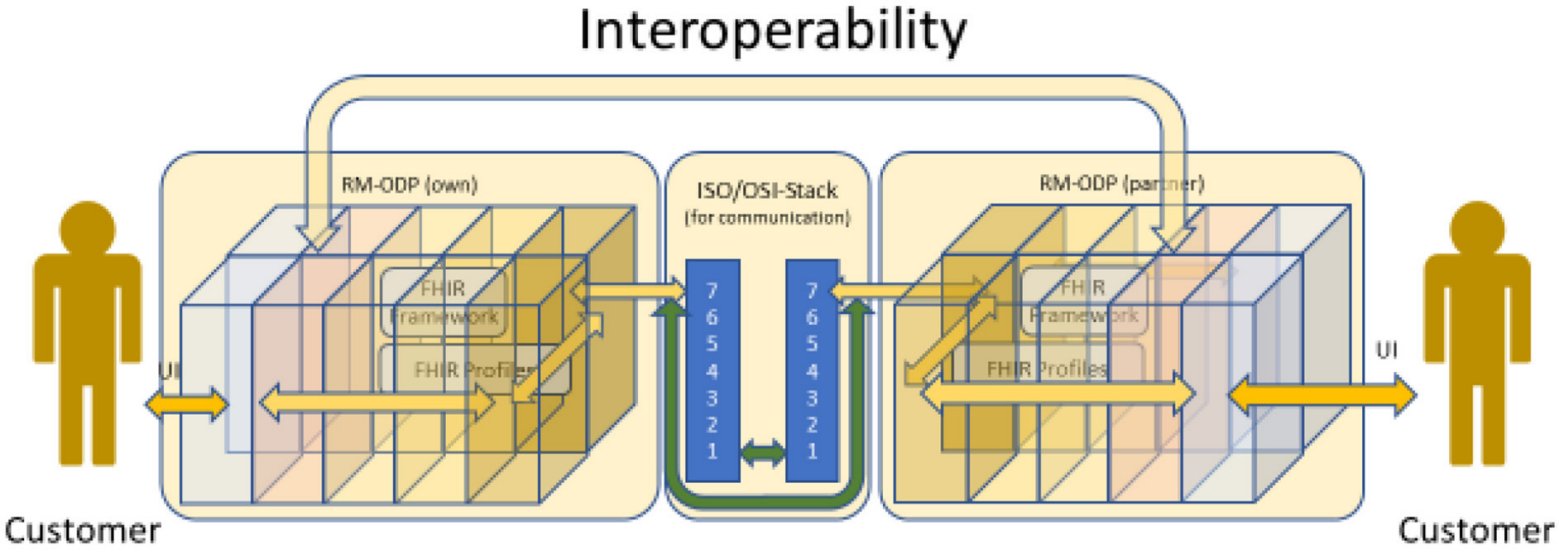
Unfolding the cube.

Using the aforementioned thoughts to reformat Figure 2 by unfolding and combining it with Figures 4, 17 results in Figure 18: The data being stored in one application according to the associated information model is communicated and transmitted to the other application and stored accordingly. Interoperable data exchange requires that the data is stored on both sides equivalently to each other, without the loss or falsification of data. Furthermore, the usage of this data has to be exactly the same. This guarantees that the concept of the business system component represented by the data is correctly understood on both sides. Without such a verification and confirmation, one can hardly name the process interoperable data exchange, although the data might be reused. For representing the business system on both sides, a generic component model (GCM) is used to represent the business system components and the related domains it serves (28).

**Figure 19:**
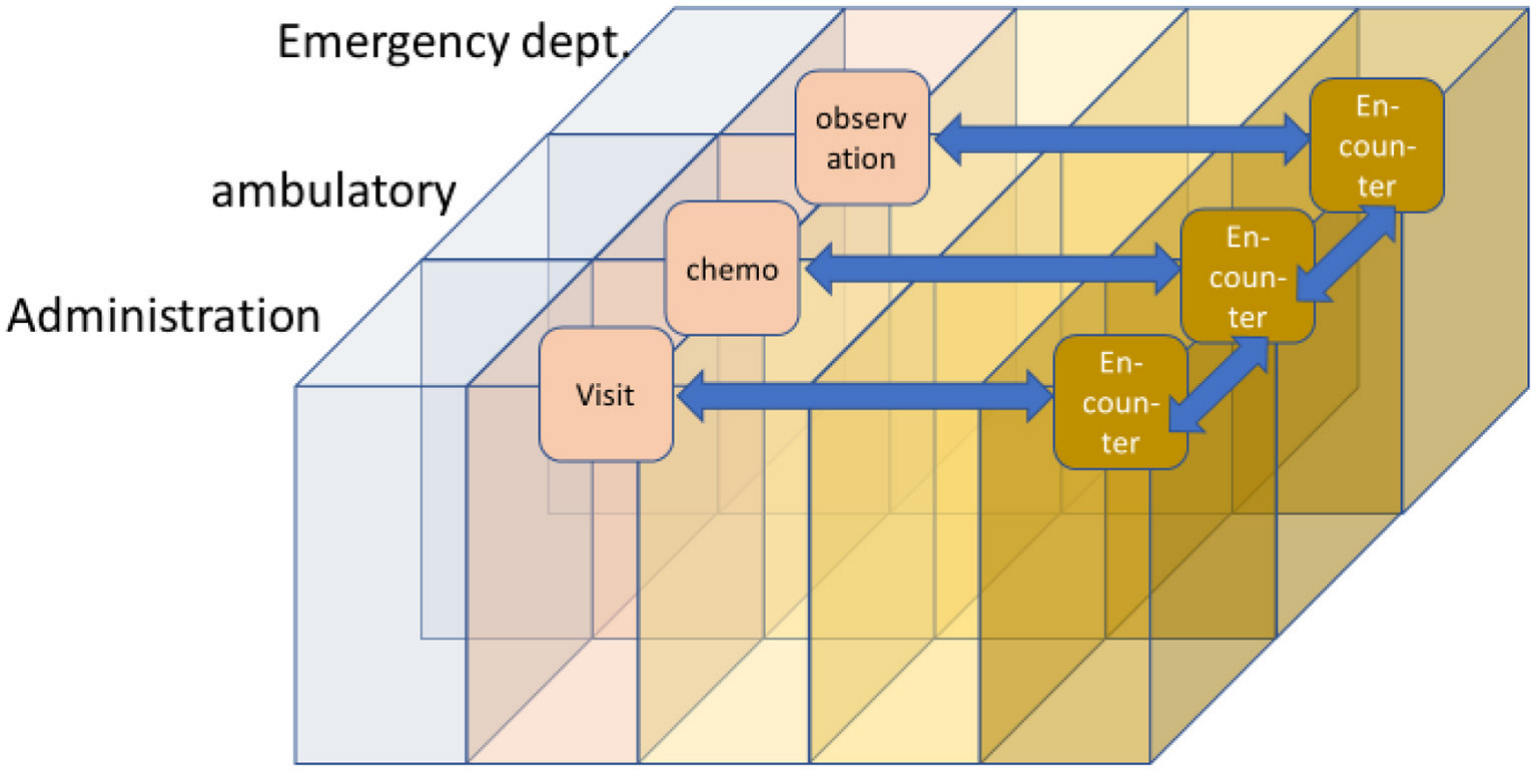
Example representation of data using the FHIR encounter resource.

Figure 19 provides an example demonstrating the difficulties using the FHIR Encounter Resource. In different domains, various types of “encounters” may occur that have to be stored with different details (attributes). Nevertheless, all of them can be communicated in FHIR using the same data structure. Therefore, there must be some clear and unambiguous indications in which way each of these communicated data has to be interpreted. Misinterpretations in any way may lead to severe risks for patient treatment.

All arguments favor information models for aligning a common understanding of data, so allowing a correct reuse.

## Model transformation

### Good modeling best practices

The different views on digital health systems discussed in the former sections are represented by different languages and different grammars. They range from business process notation languages with a grammar more constrained than natural languages, but less than the other ICT languages and ontologies, up to highly expressive traditional programming language with regular grammars, demonstrating a growing expressivity and formalization of languages. For ensuring context-sensitivity, the inclusion of tacit and implicit knowledge as well as decidability of the resulting system representation, the modeling process has to start in a top-down manner, where business domain experts define the view of the model as well as structure and naming of concepts to ensure the conceptual integrity of the model (29). Thereby, the good modeling design principles orthogonality, generality, parsimony, and propriety have to be guaranteed (30).

When modeling dynamic, multidisciplinary, transformed health ecosystems, the different perspectives of involved domains, different requirements of the intended users as well as behavioral, conceptual or contextual differences among the modelers might lead to different models of the same phenomenon. To guarantee that the integration of models represents the intended unambiguous, abstract conception of some parts or aspects of the real world, the models must be represented in an architecturally (i.e. structurally and behaviorally) correct and consistent way throughout all viewpoints. Newly created models and interrelations can only be justified at the real-world business system.

This aspect has to be considered when modeling business system components and using them for integration and/or interoperability.

### Architectural approach to model transformation

**Figure 20:**
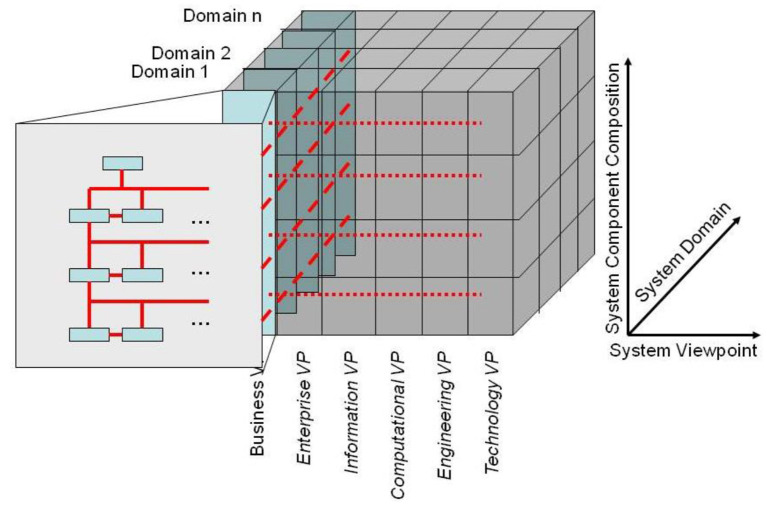
The GCM model and framework.

or overcoming the aforementioned problems, a system-oriented, architecture-centric, ontology-based, policy-driven systems representation has been developed, based on the aforementioned GCM. The resulting generic integration and interoperability reference architecture based on universal type theory, universal logics and the system of ontologies has been meanwhile standardized as ISO 23903 (31). It represents every system of systems from the perspectives of the involved domains with generic granularity levels and the system's development process according to the ISO 10746 RM-ODP (18). Using the domain ontologies, all components must be represented and interrelated in the real-world business view, and thereafter transformed into the different viewpoints (ICT models). This must be done at the same granularity level for both the interrelation of components within any viewpoint and the transformation between them (represented by the red lines in Figure 20). Details can be found in (32).

ISO/TC 215 Health Informatics as well as the related European SDO CEN/TC 251 Health Informatics have declared the deployment of ISO 23903 mandatory for any project or specification addressing multiple domains with different knowledge spaces and ontologies to represent them. That way, the correct development of new solutions as well as the integration of, or interoperability between, existing specifications can be easily performed and the correctness guaranteed. Meanwhile, ISO 23903 has been successfully used in standards specifying clinical models, presenting architectural approaches, managing concept mapping, and many more.

## Summary

The paper demonstrated that multiple aspects must be considered when designing and implementing information systems. ISO 23903 is a good basis to support the alignment of different kinds of requirements. Furthermore, it enables the mapping between different domains, different specifications and products. Therefore, it provides an universal model and framework for advanced interoperability and integration between systems and any kind of principals such as organizations, persons, devices, applications and objects. Not all of the requirements to be incorporated are controlled by just one party. This paper should have made clear that following good modeling principles acc. to ISO 23903 is a mandatory demand that challenges all participants in the system design and development process.

## Data availability statement

The original contributions presented in the study are included in the article/supplementary material, further inquiries can be directed to the corresponding author.

## Author contributions

FO planed and designed the paper and authored its major part. BB authored Section 14 and contributed to all the other sections.

## Conflict of interest

Author FO was employed by IT-Consultant in Healthcare. The remaining author declares that the research was conducted in the absence of any commercial or financial relationships that could be construed as a potential conflict of interest.

## Publisher's note

All claims expressed in this article are solely those of the authors and do not necessarily represent those of their affiliated organizations, or those of the publisher, the editors and the reviewers. Any product that may be evaluated in this article, or claim that may be made by its manufacturer, is not guaranteed or endorsed by the publisher.
